# Soft Drinks as a Dietary Source of Fluoride Exposure

**DOI:** 10.1007/s12011-023-03937-0

**Published:** 2023-11-03

**Authors:** Samuel Alejandro-Vega, Arturo Hardisson, Carmen Rubio, Ángel J. Gutiérrez, Juan R. Jaudenes-Marrero, Soraya Paz-Montelongo

**Affiliations:** 1 Area of Toxicology, Universidad de La Laguna, Canary Islands, 38071 La Laguna, Tenerife, Spain; 2 Grupo interuniversitario de Toxicología Ambiental y Seguridad de los Alimentos y Medicamentos, Universidad de La Laguna, Canary Islands, 38071 La Laguna, Tenerife, Spain

**Keywords:** Fluoride, Refreshment drinks, Soft drinks, Potentiometry, Risk assessment, Toxic risk

## Abstract

High fluoride exposures can lead to adverse effects such as dental and bone fluorosis, as well as endocrine and cognitive developmental problems. Water is the main dietary source of this ion, although significant concentrations have also been detected in other beverages widely consumed by the population such as soft drinks. A total of 200 soft drink samples (60 flavoured, 70 extracts, 60 fruit juice and 10 soft drinks) were analysed by fluoride ion selective potentiometry. A consumption of 330 mL was estimated for exposure assessment and subsequent F-risk assessment by soft drink consumption. The highest average concentration was found in extract soft drinks (2.45 ± 1.15 mg/L), followed by flavoured (1.71 ± 2.29 mg/L) and carbonated soft drinks (1.38 ± 0.40 mg/L), while the lowest was found in fruit juice soft drinks (1.09 ± 0.62 mg/L). The flavours with the highest concentration were tea-melon and tea-passion fruit with 3.66 ± 0.40 and 3.17 ± 0.56 mg/L respectively and the lowest was lemon flavour with 0.69 mg/L. The contribution of these beverages, considering the UL (Upper level) reference values set by EFSA (European Food Safety Authority) are between 3.28–41.78%, depending on age group and sex.

## Introduction

The concept of soft drink, as defined by Royal Decree 650/2011 of 9 May, is "soft drinks, carbonated or not, prepared with water for human consumption, prepared waters, natural mineral water or spring water, containing one or more of the following ingredients; carbon dioxide, sugars, …, flavourings or other food ingredients" [1]. Given the ambiguity of this description, soft drinks consist of a large amalgam of products that are very heterogeneous from one another, with the only commonality being water and sugar as the main ingredients.

Given its wide distribution and high consumption [2–4], these types of beverages have been subjected to numerous studies [5–9]. However, few studies have been carried out on their fluoride content and the corresponding toxicological risk assessment [10], although the main dietary sources of this anion include water, the main ingredient, and tea [11]. In addition, fluoride can also be found in other ingredients such as vegetable extracts [12], present in these beverages.

No adverse effects have been detected due to deficiency in fluoride intake, which is why bodies such as the EFSA do not consider this anion to be an essential element. However, dietary exposure to certain levels of fluoride may be beneficial as discussed above [11, 13]. Fluoride is incorporated into the tooth enamel during its formation, promoting its mineralisation, creating fluorohydroxyapatite, which is more resistant than the conventional hydroxyapatite structure and less vulnerable to demineralisation caused by acidogenic bacteria. This process can also occur after the enamel has been formed by topical incorporation [11, 14–17].

On the other hand, the presence of fluoride in saliva and tooth surface promotes the inhibition of different enzymatic mechanisms of acidogenic bacteria. Fluoride is not able to pass through the cell wall in its ionic form, but HF can, so when these microorganisms metabolise carbohydrates producing acids, they generate the ideal conditions for the formation of hydrofluoric acid. Once in the cytoplasm, this acid dissociates and is able to inhibit different enzymatic mechanisms, mainly glycolytic, affecting in particular the functioning of enolase and therefore reducing the production of hydrofluoric acid [11, 15, 18].

Just as dietary exposure to fluoride positively affects dental health, it also promotes better bone condition. Fluoride is one of the few agents known to stimulate osteoblast proliferation, which increases mineral deposition in these tissues. A denser bone structure makes it more resistant to fracture and degradation by osteoclasts [11, 14, 15].

However, fluoride has a hormone-like character, i.e. adequate exposure is good for health, but high exposure leads to adverse effects. The best known is dental fluorosis which is characterised by brown mottling of the teeth due to abnormal mineralisation caused by decreased protease activity and apoptosis of ameloblasts and odontoblasts [19]. This results in the appearance of gaps in the crystalline structure, increasing the porosity of the tooth and retaining part of the proteins responsible for enamel formation. Dental fluorosis occurs mainly during tooth formation and is irreversible, so special care must be taken to prevent fluoride exposure in infants from birth to 8 or 10 years of age [11, 14, 15, 20, 21]. In addition, excess fluoride in the oral cavity can lead to the formation of calcium fluoride from hydroxyapatite [22].

The most important chronic effect is bone fluorosis, however, cases in which this is attributed exclusively to dietary exposure are rare, although it has been reported in China [23]. This condition favours the deposition of calcium in the bone structure, increasing its density, as well as producing calcification of ligaments and cartilage and excessive bone growth, even leading to the union of vertebrae. Its symptoms include joint pain and limited movement, and it also hinders the recovery of fractures, due to the increased activity of calcineurin, which inhibits the functioning of osteoclasts. However, it can also lead to the development of osteoporosis and osteomalacia under conditions of lower Ca levels [21, 23, 24].

Overexposure to fluoride has been shown to lead to adverse effects on the endocrine system, such as an overactivity of the parathyroid gland, as well as an increase in alkaline phosphatase activity and calcineurin activity. Increased parathyroid hormone concentrations have been reported. This abnormal activity of the gland causes hyperplasia, degeneration and damage to the genetic material [23, 25].

It also affects both the male and female reproductive systems. In the former case through reduced oestradiol (E2) production and decreased activity of 3β-hydroxysteroid dehydrogenase (3β-HSD) and 17β-hydroxysteroid dehydrogenase (17β-HSD). In the male reproductive system, there is decreased mRNA expression of follicle stimulating hormone receptor (FSHR), luteinising hormone receptor (LHR), sex hormone binding globulin (SHBG), α-inhibin (INHα) and β-inhibin (INHβ) in the testes, as well as a reduction in the size of the testes [25].

Poor thyroid function during the early stages of life can lead to underdevelopment of the central nervous system, as has been shown in different studies correlating increased exposure to fluoride and endemic fluorosis with lower IQ in school-age populations in China, Mexico, Canada, etc. [25–30]. Prenatal exposure to high concentrations of fluoride has also been reported as a critical stage in the development of this deficiency, mainly among male individuals [24, 29]. The stage of embryogenesis is the stage of major brain development and growth, during which the proper functioning of glutamate transporter proteins, which are affected by high fluoride exposure, is crucial, and inhibition of the thyroid during this process alters early neuronal migration in the developing foetus [31].

The proper functioning of the pancreas may also be altered, as studies have reported a correlation between chronic administration of fluoride to rats and an inhibition of insulin production, which may lead to hyperglycaemia [25]. As for the adrenal gland, although few studies have been conducted, it is believed that fluoride inhibits dehydrogenase activity and cortisol production, leading to cortisaemia, which are essential for the synthesis of steroid hormones such as progesterone, glucocorticoids, mineralocorticoids, androgens and oestrogens. In addition, lesions in the proximal convoluted tubule have been reported [25, 32, 33].

It has been confirmed that fluoride generates an increase in peroxidised and radical species, leading to an increase in oxidative stress and thus causing tissue damage. This is due to the ability of this anion to bind to various enzymes, including antioxidant enzymes such as superoxide dismutase, catalase, glutathione peroxidase, glutathione reductase and superoxide dismutase, reducing their activity [34, 35]. The genotoxic potential of F- is also attributable to this oxidative stress and its ability to form covalent bonds with DNA, and this accumulation of radical species in the cytoplasm alters the proper functioning of proteins and organelles such as the endoplasmic reticulum and mitochondria [25, 35].

There is evidence that this anion can interfere with the proper functioning of mitochondria by decreasing cellular respiration and thus ATP production. The excessive presence of fluoride causes a change in membrane potential and the release of cytochrome C and induces the production of radical species in the organelle, which is the main producer of these molecules, which under normal circumstances should be eliminated [25, 35, 36].

Drinking water is the main source of fluoride in the diet, this anion is incorporated into groundwater due to the filtration of this anion by contaminated soils and mainly due to the contact of these waters with rocks and minerals that carry this anion. This contamination is favoured by different factors, such as a higher acidity and alkalinity, which allows the fluoride contained in the fluorite to be released through the formation of calcium carbonate, as well as that of the Al-F complexes through the exchange between hydroxide and fluoride ions. Volcanic soils have a low concentration of calcium, which allows a higher concentration of fluoride in the equilibrium [21, 37–40].

This is why regions such as mainland Spain have low concentrations of fluoride in drinking water, while in the Canary Islands concentrations of < LOQ to > 1.5 mg/L have been recorded, with occasional restrictions on consumption being established for part of the population [41–43]. On the other hand, a study carried out in the United Arab Emirates has detected concentrations of 0.04 to 0.28 mg/L [44], studies carried out in India show concentrations of 0.38 to 3.97 mg/L [45–48]. At the global level, concentrations found in Bangladesh (2.32 mg/L), China (14.10 mg/L), Indonesia (4.20 mg/L), Serbia (11 mg/L), Iran (9.20 mg/L), South Korea (40.8 mg/L), South Africa (15.2 mg/L), Kenya (25 mg/L), Turkey (13.7 mg/L), Norway (8.26 mg/L), Canada (15.1 mg/L) or Vietnam (28.1 mg/L) among others, also stand out [40, 49, 50].

Soft drinks, being made from various ingredients and being a product widely consumed by all population groups, could be an important source of fluoride in the diet. For these reasons, the objectives of this work are 1) to determine the fluoride concentration in soft drink samples, 2) to assess dietary fluoride exposure from soft drink consumption, and 3) to assess the risk and nutritional intake of fluoride from soft drink consumption.

## Material and Methods

### Sampling and Pre-treatment

A total of 200 samples of soft drinks of different characteristics were analysed; 60 flavoured, 70 extracts, 60 fruit juice and 10 soft drinks and flavours; 40 cola, 10 strawberry, 20 lemon-lime, 10 passion fruit, 10 peach, 30 tea-lemon, 20 tea-mango-pineapple, 30 lemon and 30 orange (Table 1).

**Table 1:** Distribution of the analysed samples

Flavoured soft drink	60
Cola	40
Strawberry	10
Lemon lime	10
Refreshing extract drink	70
Passion fruit	10
Peach	10
Lemon tea	30
Mango pineapple tea	20
Extract soft drink	60
Lemon	30
Orange	30
Carbonated soft drink	10
Lemon lime	10

The Carbonated soft drinks were treated with ultrasounds for 10 min to avoid interferences on the measure. Three aliquots were taken from each sample, in plastic containers with a 25:5 mL proportion of sample: conditioning solution.

### Fluoride Determination

Fluoride determination was performed by fluoride ion selective potentiometry using the HACH SensION-MM340 potentiometer (HACH, Düsseldorf, Germany) and the HACH ISE F-9655C fluoride ion selective electrode (HACH, Düsseldorf, Germany). The instrumental parameters are: measuring range (0.01–19000 mg/L), pH range (4–8), linear range (0.1–19,000 mg/L), slope (59 mV/pF), working temperature (5-50ºC) and possible interferences, eliminated with the conditioning solution (Fe^3+^ and Al^3+^).

For this determination it was necessary to prepare 0.75 M orthophosphoric acid as a conditioning solution [51], prepared from 85% concentrated orthophosphoric acid (Honeywell-Fluka, Germany). And a calibration line for fluoride (10^–5^, 10^–4^, 10^–3^, 10^–2^, 10^–1^ M) in the conditioning solution, prepared from NaF of analytical purity (Merck, Germany).

### Quality Control of the Method and Validation

The precision of the method was evaluated under reproducibility conditions using the standard addition method. Once the F^−^ concentration of the samples was determined, a known amount of fluoride was added. The repeatability values in terms of relative standard deviation (RSD) were 2.40% with a reproducibility of 3.20%. The recovery rate was 85.61% with an RSD of less than 12%. The values were adequate and the recovery rate was satisfactory.

### Statistical Analysis

A statistical analysis of the results obtained was carried out to determine the existence of significant differences (p < 0.05) between the different samples according to flavours and types of soft drinks. Using GraphPad Prism 9.0.2. software (GraphPad Software, USA), since the data did not follow a normal distribution, non-parametric independent variable tests (Kruskal–Wallis, Mann–Whitney) were performed.

### Calculation of Dietary Intake and Exposure Assessment

Dietary exposure and subsequent assessments were based on obtaining the estimated daily intake, EDI (Eq. 1) and its subsequent comparison with EFSA reference values (Table 2), through the percentage contribution (Eq. 2), to both the Adequate Intake and Tolerable Upper Intake Level, AI and UL respectively.

**Table 2:** Reference values established by the EFSA

Population group	Age	Sex	AI mg/day	UL mg/day
Infant	7–11 month	Both	0.4	ND
Children	1–3 years		0.6	1.5
	4–6 years	Men	1	2.5
	4–6 years	Women	0.9	
	7–8 years	Men	1.5	
	7–8 years	Women	1.4	
	9–10 years	Men	1.5	5
	9–10 years	Women	1.4	
	11–14 years	Men	2.2	
	11–14 years	Women	2.3	
Teenagers	15–17 years		3.2	7
	15–17 years		2.8	
Adults	≥ 18 years	Men	3.4	
	≥ 18 years	Women	2.9	
Pregnant	≥ 18 years			
Lactating women	≥ 18 years			

1 $$IDE=Fluoride \;concentration \left({~}^{mg}\!\left/ \!{~}_{L}\right.\right)\cdot Daily \;consumption \left({~}^{L}\!\left/ \!{~}_{day}\right.\right)$$

2 $${\%}_{Contribution}=\left(\frac{IDE\left({~}^{mg}\!\left/ \!{~}_{day}\right.\right)}{Reference \;value \left({~}^{mg}\!\left/ \!{~}_{day}\right.\right)}\right)*100$$

## Results and Discussion

### Fluoride Content

**Fig. 1:**
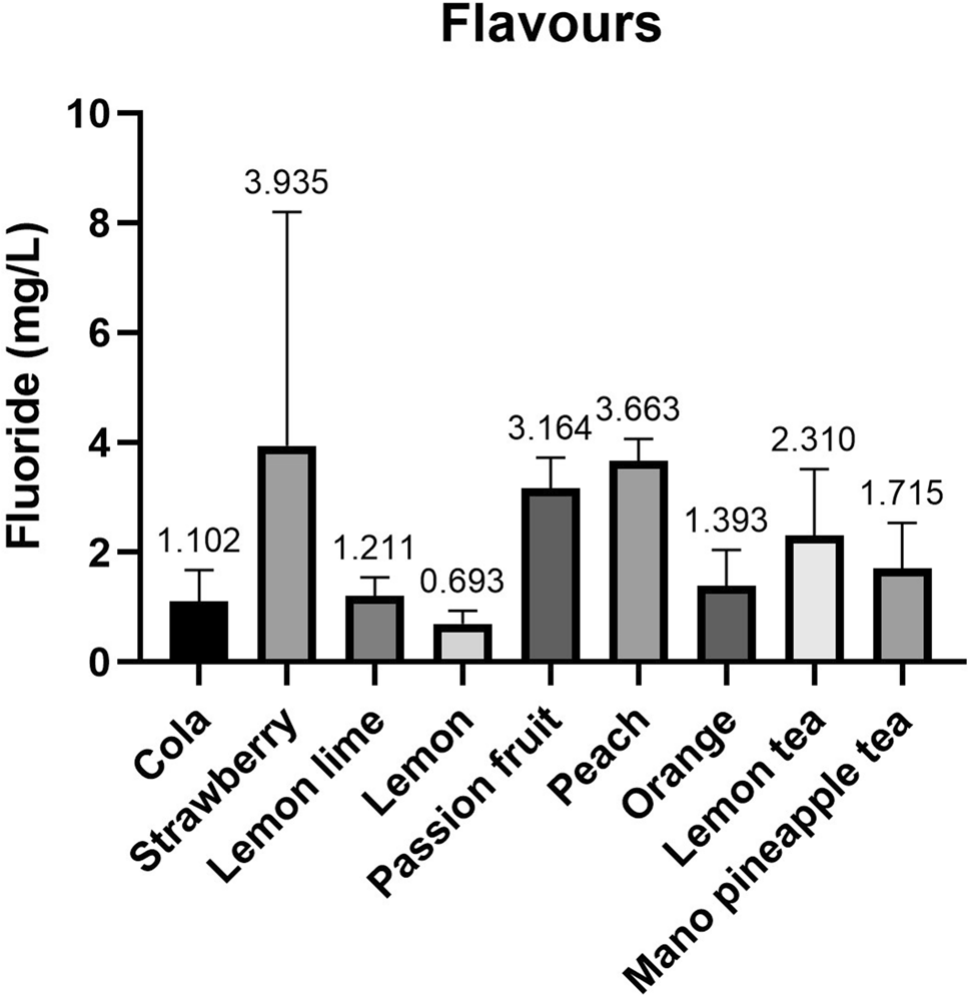
Fluoride concentration (mg/L) by flavour

**Fig. 2:**
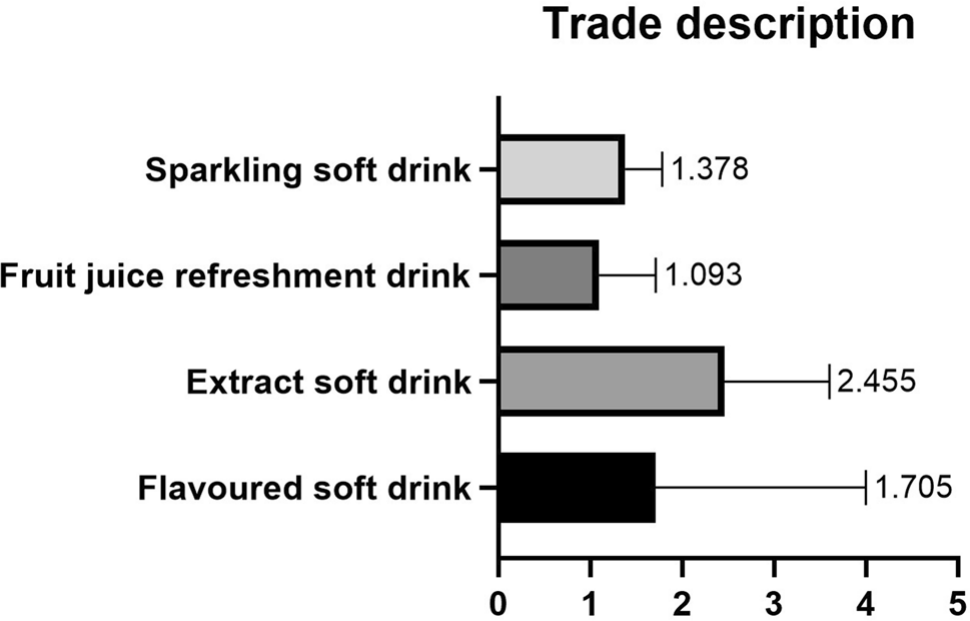
Fluoride concentration (mg/L) by trade description

The results obtained for each of the sample groups are shown in the following figures (Figs. 1 and 2). The highest fluoride concentrations were found in the strawberry flavour samples (3.94 ± 4.27 mg/L), followed by peach and passion fruit, 3.66 ± 0.40 mg/L and 3.17 ± 0.56 mg/L, respectively (Fig. 1). While the lowest concentrations were found in Lemon flavour (0.69 ± 0.24 mg/L), followed by cola and lemon-lime, 1.10 ± 0.58 mg/L and 1.21 ± 0.33 respectively, the latter considering the two categories of soft drink type where it has been studied.

Regarding the type of soft drink (Fig. 2), the highest concentration was recorded in extract soft drinks 2.45 ± 1.15 mg/L, while the lowest concentration was found in fruit juice soft drinks 1.09 ± 0.62.

These results highlight the influence of both the water used to manufacture these products and the different extracts and concentrates used. Since tea is a recognized dietary source of fluoride [11, 12, 41, 52] and the presence of concentrates of this plant in the extract-based beverages may be the reason why this category presents a higher concentration, considering that the geographical origin of the samples is diverse.

Studies on foods marketed on Tenerife Island, Spain, have found concentrations from 0.03 to 0.35 mg/L in wines[22], from 0.34 to 4 mg/L in tea [41], from 0.24 to 0.62 mg/L in bottled water [53], from 0.06 to 1.77 mg/L in beer [54], from 1.10 to 2.28 in cereals, from 0.01 to 0.99 in vegetables and from 0.32 to 1.05 in legumes [55].

While, studies of beers commercialized in the United Kingdom and Poland have found concentrations of 0.067 to 0.712 mg/L [56, 57], in Portugal concentrations of 0.40 mg/L have been found in both soft drinks and juices, as well as 0.12 and 0.16 mg/L in infusions and teas respectively [52], while in Mexico concentrations of 0.43 and 0.67 have been reported in soft drinks and juices respectively [58]. As can be seen in the present study higher concentrations have been reported in some types of soft drinks compared to the data collected by the authors previously mentioned.

### Statistical Analysis among the Beverages Analysed

The hypotheses proposed in the previous section were made taking into account the results of the different statistical analyses carried out to determine the existence, or not, of significant differences between the variables studied; flavours (Table 3) and trade description (Table 4).

**Table 3:**
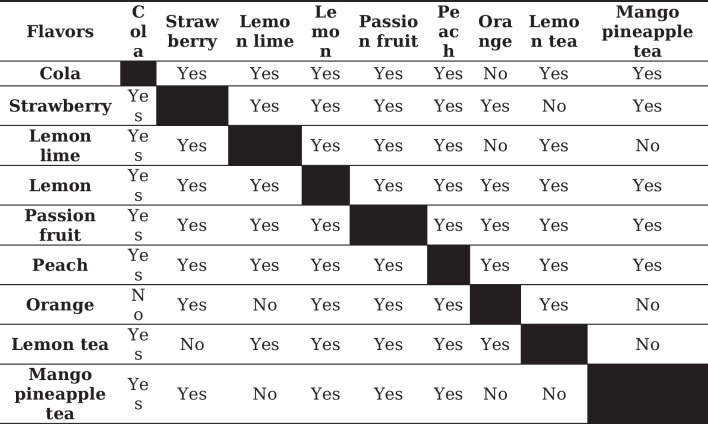
Existence or not of significant differences according to flavors

**Table 4:**
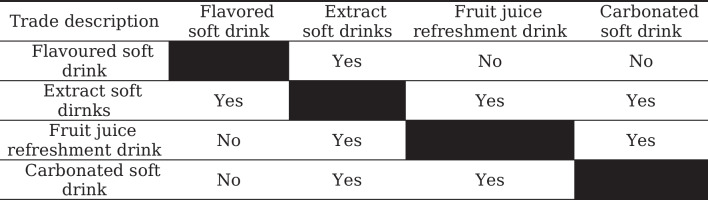
Existence or not of significant differences according to trade description

The difference between the different flavors (Table 4) emphasizes the importance not only of water, but also of tea extracts and concentrates and, to a lesser extent, those of other fruits and plants.

This influence becomes more evident when the different trade descriptions are compared. As the extract soft drinks are the only ones that contain tea and it is the only trade description with statistical differences in relation to the rest of the descriptions.

### Fluoride Intake Assessment

0.33 L volume was established as ration content. Exposure, nutritional and risk assessment was performed for the different population groups using the equations that can be found in the Materials and Methods section.

Two different consumption scenarios were considered: 1 and 2 servings. Furthermore, it should be taken into account that the overall intake (water, coffee, vegetables, marine products, …) was not evaluated, but rather the dietary exposure due to a single product. For this reason, from percentages of contribution to the UL above 15–20% there could be concern about an excess in the intake of fluoride in the diet as a whole.

#### Scenario 1: Single Ration Intake (0.33 L/day)

The estimated daily intakes (EDI) of fluoride for a ration according to flavours and trade descriptions are shown in the following table (Table 5).

**Table 5:** EDI based on flavour and trade description for 0.33 (L/day)

Flavour	EDI (mg/day)
Cola	0.36
Strawberry	1.30
Lemon lime	0.40
Lemon	0.23
Passion fruit	1.04
Peach	1.21
Orange	0.46
Lemon tea	0.76
Mango pineapple tea	0.57
Trade description	EDI (mg/day)
Flavoured soft drink	0.56
Extract soft drink	0.81
Fruit juice refreshment drink	0.36
Sparkling soft drin	0.45

In all the cases studied, these beverages, under the established conditions, can be confirmed as a possible dietary source of fluoride with a percentage of Adequate Intake higher than 7.91% (Tables 6 and 7).

**Table 6:** Contribution percentage to adequate intake established by EFSA, according to flavors, for a consumption of 0.33L/day

Population group	Age	Sex	AI mg/day	Cola	Strawberry	Lemon lime	Lemon	Passion fruit	Peach	Orange	Lemon tea	Mango pineapple tea
Infant	7–11 months	Both	0.4	90.87	324.68	99.92	57.32	261.12	302.15	114.85	190.54	141.43
Children	1–3 years	Both	0.6	60.58	216.45	66.61	38.21	174.08	201.43	76.57	127.03	94.29
	4–6 years	Men	1	36.35	129.87	39.97	22.93	104.45	120.86	45.94	76.22	56.57
	4–6 years	Women	0.9	40.39	144.30	44.41	25.48	116.06	134.29	51.04	84.69	62.86
	7–8 years	Men	1.5	24.23	86.58	26.64	15.29	69.63	80.57	30.63	50.81	37.72
	7–8 years	Women	1.4	25.96	92.77	28.55	16.38	74.61	86.33	32.81	54.44	40.41
	9–10 years	Men	1.5	24.23	86.58	26.64	15.29	69.63	80.57	30.63	50.81	37.72
	9–10 years	Women	1.4	25.96	92.77	28.55	16.38	74.61	86.33	32.81	54.44	40.41
	11–14 years	Men	2.2	16.52	59.03	18.17	10.42	47.48	54.94	20.88	34.64	25.72
	11–14 years	Female	2.3	15.80	56.47	17.38	9.97	45.41	52.55	19.97	33.14	24.60
Teenagears	15–17 years	Women	3.2	11.36	40.58	12.49	7.16	32.64	37.77	14.36	23.82	17.68
	15–17 years	Women	2.8	12.98	46.38	14.27	8.19	37.30	43.16	16.41	27.22	20.20
Adults	≥ 18 years	Men	3.4	10.69	38.20	11.75	6.74	30.72	35.55	13.51	22.42	16.64
	≥ 18 years	Women	2.9	12.53	44.78	13.78	7.91	36.02	41.68	15.84	26.28	19.51
Pregnant women	≥ 18 years	Women	2.9	12.53	44.78	13.78	7.91	36.02	41.68	15.84	26.28	19.51
Lactating women	≥ 18 years	Women	2.9	12.53	44.78	13.78	7.91	36.02	41.68	15.84	26.28	19.51

**Table 7:** Contribution percentage to adequate intake established by EFSA, according to trade description, for a consumption of 0.33L/day

Population group	Age	Sex	AI mg/day	Flavoured soft drink	Extract soft drink	Fruit juice refreshment drink	Carbonated soft drink
Infants	7–11 months	Both	0.4	140.67	202.54	90.19	113.71
Children	1–3 years	Both	0.6	93.78	135.02	60.13	75.80
	4–6 years	Men	1	56.27	81.01	36.08	45.48
	4–6 years	Women	0.9	62.52	90.02	40.09	50.54
	7–8 years	Men	1.5	37.51	54.01	24.05	30.32
	7–8 years	Women	1.4	40.19	57.87	25.77	32.49
	9–10 years	Men	1.5	37.51	54.01	24.05	30.32
	9–10 years	Women	1.4	40.19	57.87	25.77	32.49
	11–14 years	Men	2.2	25.58	36.82	16.40	20.67
	11–14 years	Female	2.3	24.46	35.22	15.69	19.78
Teenagers	15–17 years	Women	3.2	17.58	25.32	11.27	14.21
	15–17 years	Women	2.8	20.10	28.93	12.88	16.24
Adults	≥ 18 years	Men	3.4	16.55	23.83	10.61	13.38
	≥ 18 years	Women	2.9	19.40	27.94	12.44	15.68
Pregnant women	≥ 18 years	Women	2.9	19.40	27.94	12.44	15.68
Lactating women	≥ 18 years	Women	2.9	19.40	27.94	12.44	15.68

Regarding the percentages of contribution to the Tolerable Upper Intake Level (UL), as would be expected given the concentrations found, the flavors with the highest contribution are strawberry, passion fruit, peach and lemon tea (Table 8). These percentages are of concern in the population under 8 years old (> 30%) and under 14 years old, except for lemon tea (> 20%). In the remaining groups, the percentages of contribution to the UL are not relevant.

**Table 8:** Contribution percentage to the Tolerable Upper Intake Level established by EFSA, according to flavors, for a consumption of 0.33L/day

Population group	Age	Sex	UL mg/day	Cola	Strawberry	Lemon lime	Lemon	Passion fruit	Peach	Orange	Lemon tea	Mango pineapple tea
Children	1–3 years	Both	1.5	24.23	86.58	26.64	15.29	69.63	80.57	30.63	50.81	37.72
	4–6 years	Men	2.5	14.54	51.95	15.99	9.17	41.78	48.34	18.38	30.49	22.63
	4–6 years	Women	2.5	14.54	51.95	15.99	9.17	41.78	48.34	18.38	30.49	22.63
	7–8 years	Men	2.5	14.54	51.95	15.99	9.17	41.78	48.34	18.38	30.49	22.63
	7–8 years	Women	2.5	14.54	51.95	15.99	9.17	41.78	48.34	18.38	30.49	22.63
	9–10 years	Men	5	7.27	25.97	7.99	4.59	20.89	24.17	9.19	15.24	11.31
	9–10 years	Women		7.27	25.97	7.99	4.59	20.89	24.17	9.19	15.24	11.31
	11–14 years	Men		7.27	25.97	7.99	4.59	20.89	24.17	9.19	15.24	11.31
	11–14 years	Female		7.27	25.97	7.99	4.59	20.89	24.17	9.19	15.24	11.31
Teenagers	15–17 years	Women	7	5.19	18.55	5.71	3.28	14.92	17.27	6.56	10.89	8.08
	15–17 years	Women		5.19	18.55	5.71	3.28	14.92	17.27	6.56	10.89	8.08
Adults	≥ 18 years	Men		5.19	18.55	5.71	3.28	14.92	17.27	6.56	10.89	8.08
	≥ 18 years	Women		5.19	18.55	5.71	3.28	14.92	17.27	6.56	10.89	8.08
Pregnant women	≥ 18 years	Women		5.19	18.55	5.71	3.28	14.92	17.27	6.56	10.89	8.08
Lactating women	≥ 18 years	Women		5.19	18.55	5.71	3.28	14.92	17.27	6.56	10.89	8.08

As for the contributions to the UL according to the trade name (Table 9), something similar to that previously mentioned occurs, where the population under 14 years of age presents percentages that are at least close to being considered problematic. Especially in extract soft drinks, where a contribution of 54.01% has been measured for children under 3 years of age.

**Table 9:** Contribution percentage to the Tolerable Upper Intake Level established by EFSA, according to trade description, for a consumption of 0.33L/day

Population group	Age	Sex	UL mg/day	Flavoured soft drink	Extract soft drink	Fruit juice refreshment drink	Carbonated soft drink
Children	1–3 years	Both	1.5	37.51	54.01	24.05	30.32
	4–6 years	Men	2.5	22.51	32.41	14.43	18.19
	4–6 years	Women		22.51	32.41	14.43	18.19
	7–8 years	Men		22.51	32.41	14.43	18.19
	7–8 years	Women		22.51	32.41	14.43	18.19
	9–10 years	Men	5	11.25	16.20	7.22	9.10
	9–10 years	Women		11.25	16.20	7.22	9.10
	11–14 years	Men		11.25	16.20	7.22	9.10
	11–14 years	Women		11.25	16.20	7.22	9.10
Teenagers	15–17 years	Women	7	8.04	11.57	5.15	6.50
	15–17 years	Women		8.04	11.57	5.15	6.50
Adults	≥ 18 years	Men		8.04	11.57	5.15	6.50
	≥ 18 years	Women		8.04	11.57	5.15	6.50
Pregnant women	≥ 18 years	Women		8.04	11.57	5.15	6.50
Lactating women	≥ 18 years	Women		8.04	11.57	5.15	6.50

#### Scenario 2: Two Rations Intake (0.66 L/day)

The fluoride estimated daily intakes (EDI) for two rations (two cans) according to flavours and trade descriptions are shown in Table 10.

**Table 10:** EDI according to flavour and trade description for 0.66 (L/day)

Flavour	EDI (mg/day)
Cola	0.73
Strawberry	2.60
Lemon lime	0.80
Lemon	0.46
Passion fruit	2.09
Peach	2.42
Orange	0.92
Lemon tea	1.52
Mango pineapple tea	1.13
Trade description	EDI (mg/day)
Flavoured soft drink	1.13
Extract soft drink	1.62
Fruit juice refreshment drink	0.72
Carbonated soft drink	0.91

In order to avoid the overcrowding of tables, to make the document easier and clearer to read and since the first scenario proposed has shown the effectiveness of soft drinks as a nutritional source of fluoride, it has been decided to not include the tables with the percentages of contribution to AI in a consumption scenario of 0.66 L/day.

When increasing the intake, it is observed that except for the Lemon flavour, all the age groups present contribution percentages to the UL higher than 10%, with the most relevant flavors being strawberry, passion fruit, peach and lemon tea, where for all the Age groups this percentage is higher than 20%. Furthermore, Strawberry, Passion fruit and Peach exceed or are close to 100% for the under 8-year-old population (Table 11).

**Table 11:** Percentage contribution to the Tolerable Upper Intake Level established by EFSA, according to taste, for a consumption of 0.66L/day UL

Population group	Age	Sex	UL mg/day	Cola	Strawberry	Lemon lime	Lemon	Passion fruit	Peach	Orange	Lemon tea	Mango pineapple tea
Children	1–3 years	Both	1.5	48.47	173.16	53.29	30.57	139.27	161.14	61.25	101.62	75.43
	4–6 years	Men	2.5	29.08	103.90	31.97	18.34	83.56	96.69	36.75	60.97	45.26
	4–6 years	Women		29.08	103.90	31.97	18.34	83.56	96.69	36.75	60.97	45.26
	7–8 years	Men		29.08	103.90	31.97	18.34	83.56	96.69	36.75	60.97	45.26
	7–8 years	Women		29.08	103.90	31.97	18.34	83.56	96.69	36.75	60.97	45.26
	9–10 years	Men	5	14.54	51.95	15.99	9.17	41.78	48.34	18.38	30.49	22.63
	9–10 years	Women		14.54	51.95	15.99	9.17	41.78	48.34	18.38	30.49	22.63
	11–14 years	Men		14.54	51.95	15.99	9.17	41.78	48.34	18.38	30.49	22.63
	11–14 years	Women		14.54	51.95	15.99	9.17	41.78	48.34	18.38	30.49	22.63
Teenagers	15–17 years	Women	7	10.39	37.11	11.42	6.55	29.84	34.53	13.13	21.78	16.16
	15–17 years	Women		10.39	37.11	11.42	6.55	29.84	34.53	13.13	21.78	16.16
Adults	≥ 18 years	Men		10.39	37.11	11.42	6.55	29.84	34.53	13.13	21.78	16.16
	≥ 18 years	Women		10.39	37.11	11.42	6.55	29.84	34.53	13.13	21.78	16.16
Pregnant women	≥ 18 years	Women		10.39	37.11	11.42	6.55	29.84	34.53	13.13	21.78	16.16
Lactating women	≥ 18 years	Women		10.39	37.11	11.42	6.55	29.84	34.53	13.13	21.78	16.16

Regarding the trade description, the population group under 8 years old presented contribution percentages higher than 30% for all product descriptions; however, only soft drinks based on extracts presented contributions that could pose a risk for the population over 15 years old (Table 12).

**Table 12:** Percentage contribution to the Tolerable Upper Intake Level established by EFSA, according to trade name, for a consumption of 0.66L/day

Population group	Age	Sex	UL mg/day	Flavoured soft drink	Extract soft drink	Fruit juice refreshment drink	Carbonated soft drink
Children	1–3 years	Both	1.5	75.02	108.02	48.10	60.64
	4–6 years	Men	2.5	45.01	64.81	28.86	36.39
	4–6 years	Women		45.01	64.81	28.86	36.39
	7–8 years	Men		45.01	64.81	28.86	36.39
	7–8 years	Women		45.01	64.81	28.86	36.39
	9–10 years	Men	5	22.51	32.41	14.43	18.19
	9–10 years	Women		22.51	32.41	14.43	18.19
	11–14 years	Men		22.51	32.41	14.43	18.19
	11–14 years	Women		22.51	32.41	14.43	18.19
Teenagers	15–17 years	Women	7	16.08	23.15	10.31	13.00
	15–17 years	Women		16.08	23.15	10.31	13.00
Adults	≥ 18 years	Men		16.08	23.15	10.31	13.00
	≥ 18 years	Women		16.08	23.15	10.31	13.00
Pregnant women	≥ 18 years	Women		16.08	23.15	10.31	13.00
Lactating women	≥ 18 years	Women		16.08	23.15	10.31	13.00

These results are of great interest and allow us to advance in the establishment of a global intake assessment that would consider all the possible fluoride dietary sources. This is a small contribution in this collective endeavour that aims to preserve the health of citizens and improve their life quality.

## Conclusions

It has been hypothesized that the fluoride content found in soft drinks depends not only on water but is largely determined by the presence of other ingredients such as tea extracts. Extract-based soft drinks stand out for their higher fluoride concentration.

The concentrations of fluoride found in the foods analysed, and the subsequent nutritional assessment, allow us to affirm that these foods are a nutritional source of fluoride. Considering the concentrations of F^−^ found, and the risk assessment derived from these, their consumption is not recommended for population younger than 3 years old. It is advisable to moderate consumption in the population from 4 to 8 years old, especially of beverages with tea extracts. According to the obtained results, teenagers and adults are not at risk for fluoride dietary exposure from soft drink consumption, as long as they follow a responsible consumption pattern.
